# Wave Propagation in a Periodically Layered Medium, Taking into Account the Effect of Microstructure Size: A Tolerance Modelling Method

**DOI:** 10.3390/ma19163450

**Published:** 2026-08-14

**Authors:** Jarosław Jędrysiak

**Affiliations:** Department of Structural Mechanics, Łódź University of Technology, al. Politechniki 6, 90-924 Łódź, Poland; jarek@p.lodz.pl

**Keywords:** periodically layered media, periodic microstructure, microstructure size effect, tolerance modelling, wave propagations

## Abstract

The subject of consideration is a medium that is periodically layered along its thickness, *H*, in the direction of the *x*_3_ axis, resting on an undeformable base. It consists of many repeating thin layers of thickness *h* (small compared to *H*). Each layer is made up of an even number of ‘sublayers’. In the medium under consideration, it is possible to identify a small repeating element, known as *the periodicity cell*. In order to account for the influence of the size of this element (the so-called *microstructure size effect*) in the analysis of the dynamic behaviour of a periodically layered medium, *the tolerance modelling method* is applied. The derived equations have constant coefficients and take into account the microstructure size effect mainly in dynamic problems of a periodically layered medium. This effect is demonstrated using the example of wave propagation along the *x*_1_ axis, parallel to the layers, with a displacement component along the *x*_3_ axis, i.e., perpendicular to the layers. Formulas determining the wave propagation speeds in the medium under consideration are derived, and the influence of various parameters related to the medium’s structure is analysed. Furthermore, in the range of lower wave propagation speeds, the results are confirmed using the well-known asymptotic model.

## 1. Introduction

**Figure 1:**
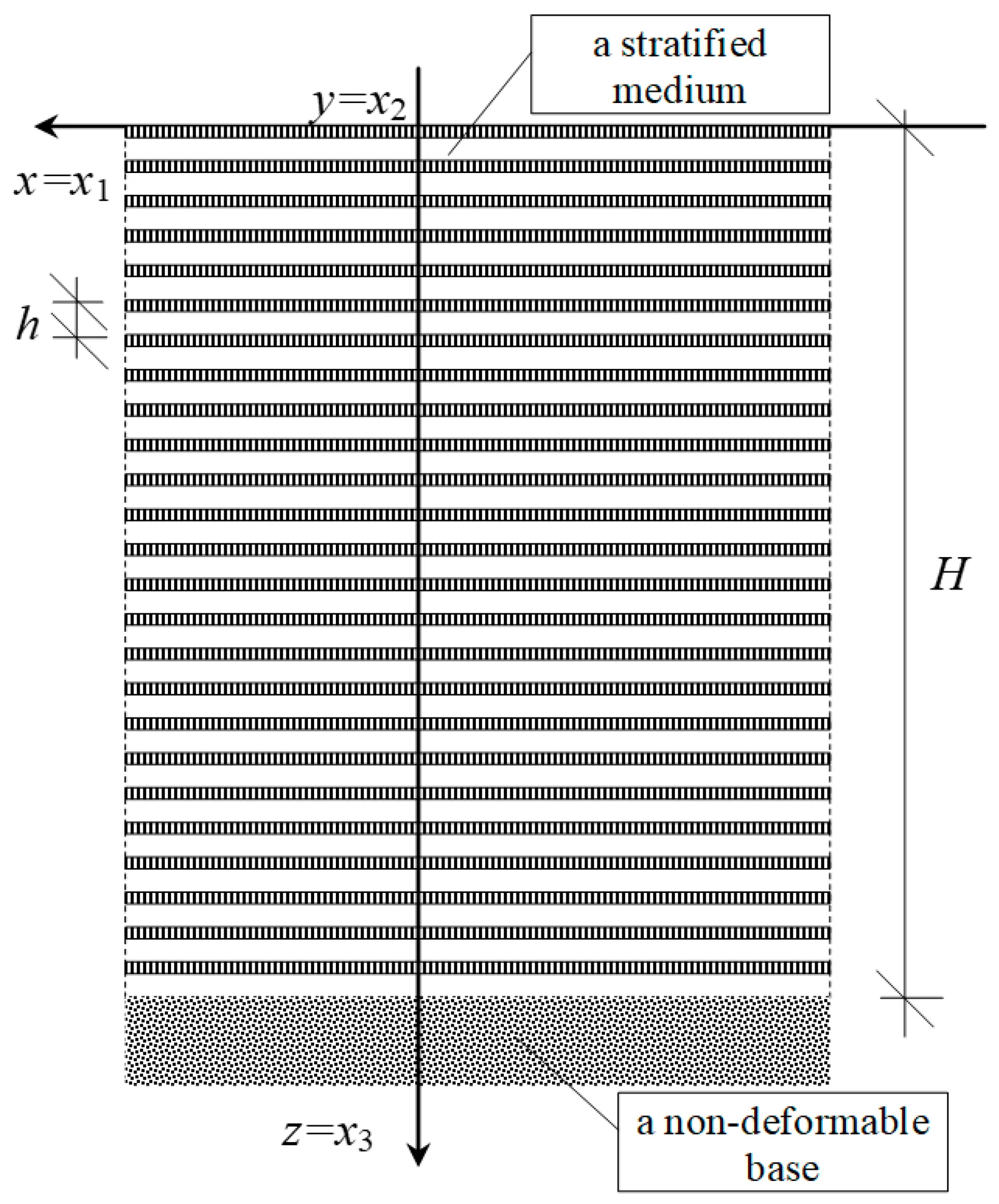
A fragment of a periodically stratified subsoil on non-deformable base.

Composites with a periodic structure, such as laminates, are used in various fields of engineering: mechanical, civil, naval, aerospace or space engineering. Their use allows for lighter and stiffer structures. This article considers composites which, at the micro level, are periodic laminates along the *x*_3_ axis, while at the macro level they are often treated as materials with a constant (averaged) distribution of properties along that axis. The subject of consideration is therefore a periodically layered medium with thickness along the *x*_3_ axis, Figure 1, resting on an undeformable base. It is assumed that its properties are periodic along the *x*_3_ axis, while constant along the *x*_1_ and *x*_2_ axes, i.e., in directions parallel to the laminates. The medium of this kind consists of many laminas periodically distributed along the *x*_3_ axis, Figure 1. Thus, it can be distinguished from a small repeated element–layer, called *a periodicity cell*. Every cell has a thickness *h* and can consist of two (or more) sub-layers (see Woźniak and Wierzbicki [1]).

Dynamics, stability and thermal problems of periodically laminated media are described by partial differential equations having functional, periodic, highly oscillating, non-continuous coefficients. These equations are not a useful tool for investigating specific problems. Therefore, various averaging methods are proposed which allow a periodic object to be replaced by a homogeneous object with constant, averaged properties. These methods convert equations with functional coefficients into equations with constant, averaged coefficients. The asymptotic procedures of homogenisation theory can be cited here [2,3,4,5]. Unfortunately, they usually lead to averaged models of periodic composites, in which *the microstructure size effect* (called also: the effect of period lengths; the length-scale effect; the dispersion phenomenon) on the behaviour of such a medium is neglected. With respect to laminated composites with a periodic structure, specialised methods have been developed. It is worth mentioning *the theories of effective stiffness* proposed and applied in [6,7]. These approaches enable the study of dispersion phenomena associated with the periodic structure of laminates. The theoretical results obtained using these methods have been verified by the experimental results presented in [8].

Various other modelling approaches have also been employed to account for the different problems associated with composite materials. Several works addressing such issues can be cited. Some of these articles, which deal mainly with various micro-inhomogeneous structures, are mentioned in this chapter. *Homogenisation with microlocal parameters* was applied to the analysis of periodic plates, see [9]; and to the modelling of certain problems related to microperiodic composite laminated half-planes, see [10,11]. In work [12], dynamic problems concerning three-layered annular plates with a viscoelastic core were investigated using two approximation methods—orthogonalisation and finite differences. Paper [13] presents the behaviour of non-classically shaped revolution shells during and after buckling, using analytical and numerical models. Similarly, works [14,15] apply analytical and numerical methods to study interesting problems related to the buckling of three-layered polyethylene plates in a magnetic field. The work [16] presents computer simulations that enabled the investigation of the effective properties and dynamic response of a laminated panel consisting of two outer sheets and an auxetic core. In [17], an analytical-numerical model is applied to study the dynamic problems caused by fluid flow in plates with different Poisson’s ratios. Work [18] utilises the finite element method to evaluate the blast resistance of sandwich panels with auxetic and non-auxetic cores. Sandwich panels having an antitetrachiral auxetic core were examined under puncture conditions [19], or under steady-state harmonic base motion [20], also applying the finite element method. The results for auxetic sandwich panels are presented in comparison with those calculated for sandwich panels with standard hexagonal honeycomb cores. In the case of composite annular structures with layers having auxetic properties, orthogonalisation and finite difference methods were used to analyse dynamic stability issues in [21].

In several articles, including [22] on composite plates with graded properties and [23] on laminated beams with a core having graded properties, mesh-free methods are applied to study natural frequencies. Meshless methods are also used in the analysis of the dynamic stability of composite laminated plates; see [24]. In the paper [25], the higher-order plate theories and the collocation method were successfully applied to the vibration analysis of composite plates with graded properties. In the paper [26], the GDQ solution for the analysis of free-vibration problems in composite shells was proposed. In [27,28], higher-order deformation theories are successfully employed to analyse thermomechanical problems in composite plates and shells with graded properties. In [29,30], the static behaviour of double-curved composite shells with graded properties is investigated. In [31], a non-classical model of a composite plate with graded properties, based on a modified couple stress theory, was presented for the analysis of thermal buckling of annular composite plates; this work also investigated the scale effect associated with couple stress theory.

In the paper [32], the natural vibrations of thick composite plates with graded properties are examined, taking into account the influence of normal and shear strains. In [33], higher-order plate theory incorporating normal and shear deformations is applied to investigate the vibrations of rectangular composite plates with graded properties. In the work [34], a strong formulation based on the GDQ technique within the finite element method is shown for multilayer plates, while [35] presents a strong formulation of isogeometric analysis for laminated composite plates. In the paper [36], the differential quadrature method and layer theory are used for composite plates. The work [37] shows an application of a new low-order shell element to shell structures with graded material properties. The differential quadrature method has been successfully applied to the analysis of a range of problems related to composite shells and plates with graded properties. For example, it is applied to determine the natural frequencies of laminated shells in [38] and to evaluate the dynamic stability of laminated shells in [39]. The same approach is also shown for the investigation of columns with open/closed cross-sections made from layered panels (see [40,41,42]). In the work [43], the natural vibrations of laminated plates with graded properties subjected to thermal loads are considered using three-dimensional finite element modelling. The authors present in the paper [44] a new analytical model for laminated structures and generalise the model to account for a continuous variation in mechanical properties in the direction of thickness of the structure wall. The individual theory of non-linear deformation of a straight line perpendicular to the neutral surface is used to develop this model. In the work [45], an analytical study is conducted on the axially symmetric bending of a generalised circular laminated plate with continuously varying mechanical properties in the direction of the core thickness, under the action of a concentrated force, with the aim of refining the theory of shear deformation. Assuming a non-linear shear deformation theory for a straight normal line in [46], an analytical model of elastic buckling of a laminated plate with a core having individual functional gradation is proposed. Numerical modelling of an annular sandwich panel with varying cladding properties is presented in the paper [47], which compares the results obtained using the finite difference method and the finite element method. In some works [48,49], a generalised differential quadrature method is applied to model the vibrations of nanocomposite shells with graded properties. In the paper [50], a highly accurate and natural model is proposed for the static mechanical analysis of annular structures with graded properties under pressure, with arbitrary elastic properties in the radial direction.

However, it should be noted that the model equations derived within the proposed modelling approaches for microstructural media do not usually take into account the influence of microstructure size. On the other hand, this effect may be of importance in the context of vibrations in these media. As Brillouin [51] noted, there are relations between macro- and micro-vibrations, the first referring to the macrostructure and the second to the microstructure, respectively. To address similar problems, specific methodologies taking this effect into account have been employed. This consideration has been extended to periodic structures in several studies, some of which are cited in this article. The theories of effective stiffness used for periodic laminates in [6,7] allow for analysing the dispersion phenomena related to the periodic structure of laminates. These results have been verified by the experimental results shown in [8]. The characteristics of band gaps in Mindlin’s periodic plates are analysed in [52], using the spectral element method. In the works [53,54], band gaps in thin periodic plates with and without damping are investigated applying the centre-point finite difference method. However, band gaps in bending waves in periodic composite plates are considered using the quadrature difference method in [55].

*The tolerance modelling method* (or *tolerance method*) provides an alternative approach to the study of various mechanical problems relating to microstructural media (periodic and non-periodic). More details can be found in the publications in [56,57]. This method is useful for a wide range of problems described by differential equations with highly oscillating, discontinuous functional coefficients. The modelling procedure based on this approach replaces the exact governing equations with averaged model equations that have constant (or slowly varying) coefficients. Some of these coefficients depend explicitly on the microstructure size.

This method allows for the investigation of a range of issues relating to dynamics, stability and thermoelasticity in periodic structures, as described in a series of articles. The tolerance method was used to investigate dynamic phenomena in microperiodically layered media [58,59]. Interesting examples of this approach involve the analysis of fluid-saturated periodic substrates, as presented in [60]. The dynamics of periodic plane structures were explored in [61]. The application of this method to the vibrations of periodic plates of medium thickness was discussed in [62,63]. An analysis of vibrations of thin unidirectional periodic plates with a thickness less than the period cell length was described in [64]. The vibrations of corrugated periodic plates were analysed in [65]. The dynamics of thin periodic plates reinforced with stiffeners was presented in [66]. Study [67] analysed the dynamics of thin periodic plates with microstructure size on the order of the plate thickness. Wave propagation in multiperiodic fibre-reinforced composites was considered in [68]. The works [69,70,71] show the application of this method to various thermomechanical problems, including dynamics and stability, for thin cylindrical shells with bidirectional or unidirectional micro-periodic structures. Paper [72] examines the behaviour of thin periodic plates under conditions of geometric non-linearity. The article [73] proposes a specific tolerance model for vibrations of periodic three-layer plates, while the work [74] compares various dynamic models. An analytical model of vibrations of a three-layered sandwich plate with a honeycomb core based on the tolerance method is presented and discussed in [75]. Problems of dynamics and stability of visco-elastic periodic slender beams on a damping foundation are considered in [76]. The problem of thermal conductivity in a periodic laminate, taking into account uncertainties in material properties, was discussed in [77,78]. Referring to [79,80], a tolerance method combined with a finite difference algorithm was used to investigate the heat conduction in bi-periodic or [81] periodically laminated composites with third-type boundary conditions. Heat conduction in periodic laminates was also analysed in [82], which compared results obtained using the tolerance method with those from the FEM and experimental results; and in [83], which analysed shape functions. In the paper [84], a multi-scale study of the stress distribution in thin periodic composite plates was shown. The analytical analysis of the such plates within the tolerance method was also performed for vibrations in [85] and for stability in [86].

Using the tolerance method, it has also been possible to successfully model materials with functional gradation and non-periodic microstructure. Dynamic issues regarding laminates with functionally graded properties were considered in [87,88]. Works [89,90] analysed the thermoelasticity of laminates with transverse gradation, taking into account the effect of microstructure size. The dynamical problem of a functionally graded laminated medium is considered in [91]. The vibration behaviour of plates with longitudinal gradation was analysed in [92,93], while the stability of similar plates was studied in [94]. The authors in [95,96] applied tolerance-based dynamic models to thin-walled structures with a dense rib arrangement. Heat transfer in cylindrical composite conductors with a non-uniform distribution of constituents was presented in [97,98]. Further studies of heat conduction phenomena in laminates with functionally graded material properties were carried out in [99], using third-type boundary conditions. The vibrations of thin plates with in-plane transverse gradation, whose thickness is smaller than the microstructure size, were presented in [56,100,101]. In the paper [102], the tolerance method was used to describe the vibrations of medium-thickness plates, characterised by a gradual change in properties across their cross-section and with a microstructure. Dynamic problems of thin shells with a microstructure with functional gradation were discussed in [103,104,105], and their dynamics and stability were analysed in [106]. It should be noted, however, that these papers do not discuss all the issues that have so far been addressed in the literature on the tolerance modelling method for microstructured media; consequently, they do not contain a comprehensive overview of this field.

The aim of this paper is to propose a tolerance model for wave propagation in a periodically laminated medium resting on a rigid substrate, and subsequently to apply the model equations in the analysis of transverse wave propagation along the *x*_1_-axis with displacement component along the *x*_3_ axis. The tolerance model made it possible to determine formulas for two transverse wave propagation velocities—the fundamental lower one, related to the macrostructure of the medium, and the higher one, related to the microstructure of the medium. The values of the lower speeds were compared with the values obtained according to the known asymptotic model, which allows only the lower speed of transverse wave propagation to be determined.

## 2. Theoretical Background

### 2.1. Modelling Preliminaries

Let subscripts *i*, *j*, …, related to the system of coordinates *Ox*_1_*x*_2_*x*_3_, run over 1, 2, 3; and subscripts α, β, …, (being related to *Ox*_1_*x*_2_) run over 1, 2; superscripts *A*, *B*, … run over 1,…,*N*. Summation convention holds for all aforementioned indices. Let: **x** ≡ (*x*_1_,*x*_2_), *x* ≡ *x*_1_, *z* ≡ *x*_3_; and *t* stands for the time coordinate. Derivatives with respect to *x_i_*, *x*_α_, *z* can be denoted by ∂*_i_*, ∂_α_, and ∂, respectively (Table 1).

A laminated subsoil occupies the region Ω ≡ (0,*H*) × Π, where Π is a region on the plane 0*x*_1_*x*_2_; *H* is the medium thickness along the *z*-axis. This laminated subsoil under consideration is made of two components distributed in laminas with the same thickness *h*. It is assumed that condition *h* << *H* is satisfied. Hence, thickness *h* can be called *the microstructure size parameter*. By Δ ≡ (−*h*/2,*h*/2) × {**0**}, the periodicity cell of a periodic medium can be denoted. Every lamina consists of two homogeneous sub-laminas with thicknesses *h*′, *h*″, cf. Figure 1. The sub-laminas have properties described by: mass densities ρ′, ρ″; elasticity tensors with components ${c^{\prime }}_{ijkl},\;{c^{\prime \prime }}_{ijkl}$; where *i*,*j*,*k*,*l* = 1,2,3. Hence, it is assumed that all inertial and material properties of the subsoil, i.e., a mass density ρ = ρ(*z*) and elastic moduli *c_ijkl_* = *c_ijkl_*(*z*), are highly oscillating, periodic, non-continuous functions in *z* and independent of **x**. Let ν′, ν″ be material volume fractions in laminas, defined as ν′ = *h*′/*h*, ν″ = *h*″/*h*, which satisfy the condition ν′ + ν″ = 1. Hence, a constant parameter γ = ν′ can be introduced, called *the non-homogeneity ratio*.

Moreover, let *u_i_*, *e_ij_*, *s_ij_* be displacements, deformations, and stresses, respectively; *p_i_* = *p_i_*(**x**,*t*) be loadings in the *x_i_* axis directions on the upper surface Π of the subsoil. Using the known relations of elastic solid mechanics, after some manipulations, the system of governing equations is derived:

(1) $$\partial_{j}(c_{ijkl}\partial_{l}u_{k})-\rho {\ddot{u}}_{i}=-p_{i},$$

which is the known system of partial differential equations for laminates and has coefficients that are non-continuous, highly oscillating, periodic functions in *z* = *x*_3_.

Because Equation (1) is not a good tool to investigate dynamic problems of periodically stratified subsoil, these equations are usually replaced by differential equations with smooth, constant coefficients. Here, in order to obtain averaged equations, *the tolerance modelling method* will be applied, cf. the book [57].

### 2.2. Tolerance Modelling Background

#### 2.2.1. Basic Concepts of Tolerance Modelling

In the tolerance modelling method, some basic concepts are used, which were formulated in the general form in the books [1,56,57]. Below, some of them are presented for the paper to be self-consistent.

Remind that *x* = *x*_1_, *y* = *x*_2_ and *z* = *x*_3_; denote an interval along the *z*-axis by Λ = (0,*H*).

Denoting a cell at *z* ∈ Λ_Δ_ by Δ(*z*) ≡ *z* + Δ, Λ_Δ_ = {*z* ∈ Λ: Δ(*z*) ⊂ Λ}, for an integrable function *f*, the *averaging operator* is defined by:

(2) $$<f>(z)=h^{-1}\int_{\Delta (z)}f(\zeta )d\zeta ,\;z\in \Lambda ,\;\zeta \in \Delta (z).$$

For a periodic function *f*, the averaged value calculated from (2) is constant.

Let ∂*^k^*θ be the *k*-th gradient of function θ = θ(*z*), *z* ∈ Λ, *k* = 0,1, $\partial^{0}\theta \equiv \theta$ and let ${\tilde{\theta }}^{\left(k\right)}(\cdot ,\cdot )$ be a function defined in $\bar{\Lambda }\times R^{m}$. Denote by χ the small parameter (χ << 1), called *the tolerance parameter*, related to every analysed problem.

*The tolerance-periodic function* $\theta \in H^{k}(\Lambda )$, $\theta \in TP^{k}(\Delta ,\chi )$, for *k* = 0,1, holds the conditions

(a) (∀z∈Λ) (∃θ˜(k)(z,⋅)∈H0(Λ)) [||∂kθΛΔ(⋅)−θ˜(k)(z,⋅)||H0(Λ,Δ)≤χ],(b) ∫Δ(⋅)θ˜(k)(⋅,ζ)dζ∈C0(Λ¯).

Function ${\tilde{\theta }}^{\left(k\right)}(z,\cdot )$ is the *periodic approximation of* $\partial^{k}\theta$ *in* Δ(z), z∈Λ, k=0,1.

*The slowly varying function*, $\Xi \in SV^{1}(\Delta ,\chi )$, is a function $\Xi \in H^{1}(\Lambda )$ satisfying

$$\begin{array}{l} (a)\;\Xi \in TP^{1}(\Delta ,\chi ),\\ (b)\;(\forall z\in \Lambda )\;[{\tilde{\Xi }}^{\left(k\right)}(z,\cdot ){|}_{\Delta (z)}=\partial^{k}\Xi (z),\;k=0,1]. \end{array}$$

Periodic approximation ${\tilde{\Xi }}^{\left(k\right)}$ of ∂*^k^*Ξ in Δ(z) is a constant function for every *z* ∈ Λ.

*The highly oscillating function*, $\vartheta \in HO^{1}(\Delta ,\chi )$, is a function $\vartheta \in H^{1}(\Lambda )$ if

$$\begin{array}{l} (a)\;\vartheta \in TP^{1}(\Delta ,\chi ),\\ (b)\;(\forall z\in \Lambda )\;[{\tilde{\vartheta }}^{\left(k\right)}(z,\cdot ){|}_{\Delta (z)}=\partial^{k}\tilde{\vartheta }(z),\;k=0,1],\\ (c)\;\forall \;\Xi \in SV^{1}(\Delta ,\chi )\;\exists f\equiv \vartheta \Xi \in TP^{1}(\Delta ,\chi )\;\;{\tilde{f}}^{\left(k\right)}(z,\cdot ){|}_{\Delta (z)}=\Xi (z)\partial^{k}\tilde{\vartheta }(z){|}_{\Delta (x)},\;k=1. \end{array}$$

Let $\tilde{f}\equiv {\tilde{f}}^{\left(0\right)}$ be denoted for *k* = 0.

Let us introduce highly oscillating functions *g*(·),$g\in HO^{1}(\Delta ,\chi )$. They are determined on $\bar{\Lambda }$ and have piecewise continuous and bounded gradients ∂^1^*g*. Functions *g*(·) are *the fluctuation shape functions* of the 1st kind, $g\in FS^{1}(\Delta ,\chi )$, if they satisfy conditions:

(*a*) ∂*^k^g* ∈ *O*(*h^s^*^−*k*^)   for *k* = 0,1, *s* = 1, ∂^0^*g* ≡ *g*, (*b*) <ρ*g*>(*z*) ≈ 0   for every *z* Λ Λ_Δ_,

and depend on the microstructure parameter *h*; ρ > 0 is a certain periodic function.

#### 2.2.2. Tolerance Method Assumptions

The tolerance method is based on some modelling assumptions, which were formulated in a general form in the books [1,56,57]. For special issues of different structures, they were adopted in many papers using basic concepts. Now, for the considered periodically stratified subsoil these assumptions are reminded.

*The micro–macro decomposition* is the first assumption, in which displacements of the subsoil are assumed as:

*u_i_*(**x**,*z*,*t*) = *W_i_*(**x**,*z*,*t*) + *g*(*z*)*V_i_*(**x**,*z*,*t*), (3)

with functions *W_i_*(**x**,·,*t*) and *V_i_* (**x**,·,*t*) being new basic kinematic unknowns, named *the macrodisplacements* and *the fluctuation amplitudes*, respectively. It is assumed that they are slowly varying functions of *z*, i.e., *W_i_*(**x**,·,*t*), *V_i_*(**x**,·,*t*) ∈ *SV*^1^(Δ,χ).

Because the object under consideration is the medium on the non-deformable foundation, it is assumed that the displacements *u_i_*(**x**,*z*,*t*) along the *z* axis are described by the known *decay function* φ(*z*), satisfying the conditions φ(0) = 1, φ(*H*) = 0, φ_,3_ < 0, cf. [107]. Hence, functions *W_i_*(**x**,·,*t*) and *V_i_* (**x**,·,*t*) can be rewritten in the form:

*W_i_*(**x**,*z*,*t*)=φ(*z*)*w_i_*(**x**,*t*)*, V_i_*(**x**,*z*,*t*)=φ(*z*)*v_i_*(**x**,*t*),

where *w_i_*(**x**,*t*)*, v_i_*(**x**,*t*) are new unknowns, which are independent on *z*.

On the other hand, function *g*(·) is the known fluctuation shape function. It is assumed *a priori* in that problem and determines the displacement *u_i_* oscillations caused by a periodic microstructure of the subsoil. Following the book [56], the fluctuation shape function, *g*(*z*), $z\in \bar{\Lambda }$, is defined to be continuous, linear across every sub-lamina thickness, and of an order *O*(*h*). Moreover, they have to satisfy the pertinent conditions (*a*)–(*b*), i.e., they can be written as:

(4) $$g(z)=\left\{\begin{array}{l} -h\sqrt{3}\frac{1}{1-\gamma }((2\frac{z}{h}+\gamma )\;\;\;\mathrm{for}\;\;\;\;z\in (-h/2,-h/2+h(1-\gamma )),\\ h\sqrt{3}\frac{1}{\gamma }(2\frac{z}{h}-1+\gamma )\;\;\;\;\;\mathrm{for}\;\;\;\;z\in (h/2-h\gamma ,h/2). \end{array}\right.$$

**Figure 2:**
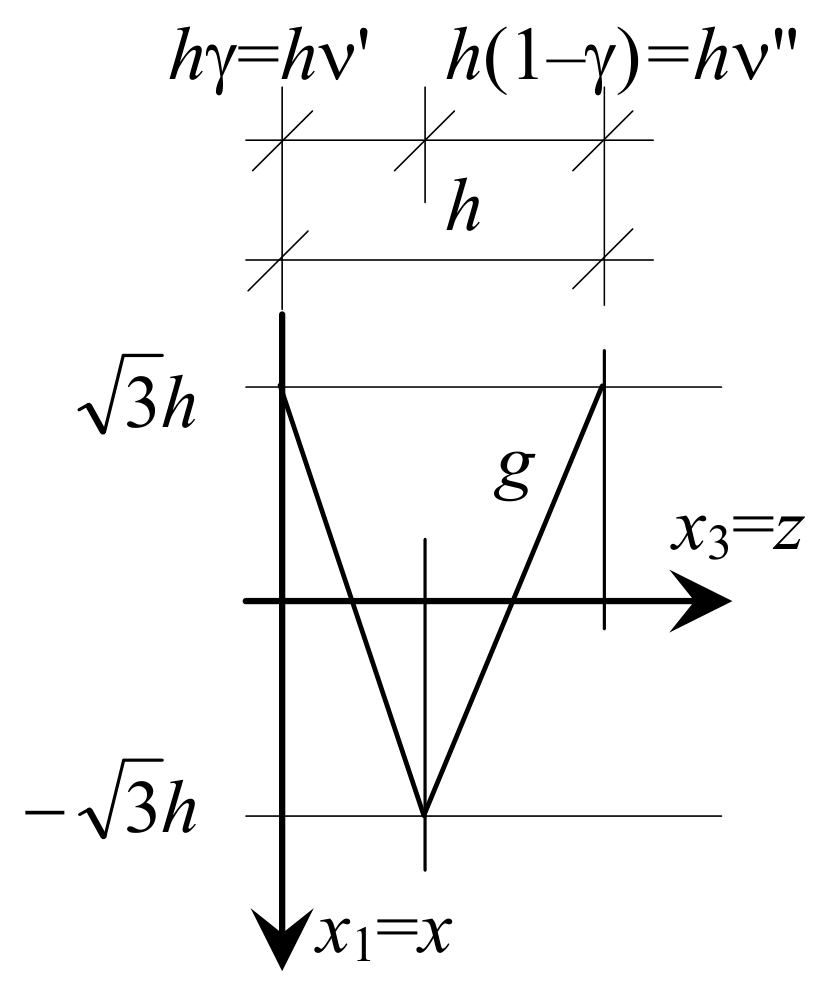
Fluctuation shape function.

The mean values of *g* in laminas are equal to zero. An example of this function is shown in Figure 2.

*The tolerance averaging approximation* is the next assumption*,* which determines that terms *O*(χ) are negligibly small, e.g., for θ ∈ *TP*^1^(Δ,χ), Ξ ∈ *SV*^1^(Δ,χ), *g* ∈ *FS*^1^(Δ,χ), in:

(5) $$\begin{array}{l} <\theta >(z)=<\bar{\theta }>(z)+O(\chi ),\\ <\theta \Xi >(z)=<\theta >(z)\Xi (z)+O(\chi ),\\ <\theta \partial (g\Xi )>(z)=<\theta \partial g>(z)\Xi (z)+O(\chi ). \end{array}$$

#### 2.2.3. Tolerance Modelling Procedure

In the books [1,56,57], the tolerance modelling procedure was presented in a different form. The procedure is shortly briefed below to make the work self-consistent, similarly to that in [91]. In the first step of the tolerance modelling procedure, the micro–macro decomposition (3) is substituted into Equation (1). Because after this Equation (1) does not hold, a residual field *r_i_*(·) exists within the macro-dynamics, and is defined in the following form:

(6) $$r_{i}=\partial_{j}(c_{ijkl}\partial_{l}u_{k})-\rho {\ddot{u}}_{i}+p_{i},$$

In *the residual orthogonality condition*, being the second step of the procedure, the residual field *r_i_*(·) is assumed to satisfy the conditions:

(7) $$<r_{i}>(x,z,t)=0,\;\;\;\;\;\;\;<r_{i}g>(x,z,t)=0.$$

A system of governing equations for the components (of the macro-displacements) *w_i_*(**x**,*t*) and the components (the fluctuation amplitudes) *v_i_*(**x**,*t*) will be obtained after applying the conditions (7), combined together with the modelling assumptions.

## 3. Averaged Governing Equations

### 3.1. Equations of Tolerance Model

The residual orthogonality conditions (7), combined with the tolerance modelling assumptions after some manipulations, lead to the averaged governing equations of the considered periodically stratified medium on a foundation for *w_i_*(**x**,*t*) and *v_i_*(**x**,*t*). Introducing denotations of certain coefficients related to the decay function φ(*z*):

(8) $$d\equiv \int_{0}^{H}\varphi^{2}dz,\;\;\;\;d_{3}\equiv \int_{0}^{H}\varphi \partial \varphi dz,\;\;\;\;d_{33}\equiv \int_{0}^{H}{\left(\partial \varphi \right)}^{2}dz,$$

*the tolerance model equations of a periodically stratified medium on a non-deformable foundation* can be written as:

(9) $$\begin{array}{l} -\partial_{\alpha }(d<c_{\alpha \beta \gamma \delta }>\partial_{\delta }w_{\gamma }+d_{3}<c_{\alpha \beta 33}>w_{3}+d<a_{\alpha \beta 33}\partial g>v_{3})+\\ +(d_{3}<c_{\beta 3\alpha 3}>\partial_{\alpha }w_{3}+d_{33}<c_{\beta 3\alpha 3}>w_{\alpha }+d_{3}<c_{\beta 3\alpha 3}\partial g>v_{\alpha })+\\ +d<\rho >{\ddot{w}}_{\beta }=p_{\beta },\\ -\partial_{\alpha }(d<c_{\alpha 33\beta }>\partial_{\beta }w_{3}+d_{3}<c_{\alpha 3\beta 3}>w_{\beta }+d<c_{\alpha 3\beta 3}\partial g>v_{\beta })+\\ +(d_{3}<c_{\alpha \beta 33}>\partial_{\alpha }w_{\beta }+d_{33}<c_{3333}>w_{3}+d_{3}<c_{3333}\partial g>v_{3})+\\ +d<\rho >{\ddot{w}}_{3}=p_{3},\\ \underline{d<\rho gg>{\ddot{v}}_{\beta }}-d\underline{<c_{\alpha \beta \gamma \delta }gg>}\partial_{\alpha \delta }v_{\gamma }+\\ +d<c_{\alpha 3\beta 3}\partial g>\partial_{\beta }w_{3}+d_{3}<c_{\alpha 3\beta 3}\partial g>w_{\beta }+d<c_{\alpha 3\beta 3}\partial g\partial g>v_{\beta }=0,\\ \underline{d<\rho gg>{\ddot{v}}_{3}}-d\underline{<c_{\alpha 3\beta 3}gg>}\partial_{\alpha \beta }v_{3}+\\ +d<c_{\alpha \beta 33}\partial g>\partial_{\alpha }w_{\beta }+d_{3}<c_{3333}\partial g>w_{3}+d<c_{3333}\partial g\partial g>v_{3}=0, \end{array}$$

where α, β = 1, 2; which include the underlined coefficients dependent on the microstructure size parameter *h*, being the thickness of laminas. Hence, the microstructure size effect is described by Equation (9) on the overall dynamic behaviour of the considered stratified subsoils/media.

### 3.2. Equations of Asymptotic Model

The above model, which is derived by applying the tolerance modelling method, makes it possible to calculate some results. These results can be evaluated by the results from the approximate model. The governing equations of this approximate model eliminate the microstructure size effect. The proper asymptotic modelling procedure leading to such equations was presented in [56] and other works, e.g., [89,108]. However, these equations can also be derived directly from the tolerance model Equation (9) by eliminating the underlined coefficients, which depend on the microstructure size parameter *h*. These equations take the following form:

(10) $$\begin{array}{l} -\partial_{\alpha }(d<c_{\alpha \beta \gamma \delta }>\partial_{\delta }w_{\gamma }+d_{3}<c_{\alpha \beta 33}>w_{3}+d<a_{\alpha \beta 33}\partial g>v_{3})+\\ +(d_{3}<c_{\beta 3\alpha 3}>\partial_{\alpha }w_{3}+d_{33}<c_{\beta 3\alpha 3}>w_{\alpha }+d_{3}<c_{\beta 3\alpha 3}\partial g>v_{\alpha })+d<\rho >{\ddot{w}}_{\beta }=p_{\beta },\\ -\partial_{\alpha }(d<c_{\alpha 33\beta }>\partial_{\beta }w_{3}+d_{3}<c_{\alpha 3\beta 3}>w_{\beta }+d<c_{\alpha 3\beta 3}\partial g>v_{\beta })+\\ +(d_{3}<c_{\alpha \beta 33}>\partial_{\alpha }w_{\beta }+d_{33}<c_{3333}>w_{3}+d_{3}<c_{3333}\partial g>v_{3})+d<\rho >{\ddot{w}}_{3}=p_{3},\\ d<c_{\alpha 3\beta 3}\partial g>\partial_{\beta }w_{3}+d_{3}<c_{\alpha 3\beta 3}\partial g>w_{\beta }+d<c_{\alpha 3\beta 3}\partial g\partial g>v_{\beta }=0,\\ d<c_{\alpha \beta 33}\partial g>\partial_{\alpha }w_{\beta }+d_{3}<c_{3333}\partial g>w_{3}+d<c_{3333}\partial g\partial g>v_{3}=0. \end{array}$$

Equation (10) stands for *the asymptotic model equations of a periodically stratified medium on a non-deformable foundation*, eliminating the microstructure size effect on the overall behaviour of the stratified subsoils. Coefficients in the above equations are constant, similarly to Equation (9) for the tolerance model, but in contrast to Equation (1), have highly oscillating, periodic, non-continuous coefficients.

## 4. An Application: A Transverse Wave Propagation Along the *x*_1_ = *x* Axis

### 4.1. Tolerance Model

#### 4.1.1. Preliminaries

As an example of application, let us consider a problem of transverse wave propagation in periodically stratified subsoil being made of two constituents. Every repeated cell (layer) has the thickness *h* and is assumed to consist of two homogeneous sub-layers having thicknesses *h*′, *h*″ (*h*′ + *h*″ = *h*). The subsoil is assumed to be made of isotropic materials. Let λ, μ be the Lame moduli, taking the constant values λ′, μ′ and λ″, μ″ in the sub-layers. Similarly, the mass density ρ is equal to ρ′ and ρ″, respectively.

In this example, it is assumed that the components of displacements *u*_α_, α = 1, 2, along the *x*_1_ and the *x*_2_ axes are equal to zero. Therefore, the relevant components *w*_α_ and *v*_α_ are also neglected. Only the component of displacement *u*_3_ along the *x*_3_ axis is not zero, and so the component *w*_3_ and the fluctuation amplitude *v*_3_ are also non-zero. Let us therefore denote *w* = *w*_3_ and *v* = *v*_3_. The wave propagation in the unbounded subsoil along the *x*_1_ = *x*-axis is investigated. Hence, loadings *p_i_*, *i* = 1,…3, are eliminated.

#### 4.1.2. Model Equations for Transverse One-Directional Wave Propagation

Using the Lame moduli, it can be written:

$$\begin{array}{l} a_{1111}=a_{2222}=a_{3333}=\lambda +2\mu \equiv \kappa ,\\ a_{1122}=a_{1133}=a_{2233}=\lambda ,\;\;\;\;\;\;\;\;\;\;\;\;\;\;\;\;\;\;\;a_{1212}=a_{1313}=a_{2323}=\mu , \end{array}$$

Equation (9) takes the form:

(11) $$\begin{array}{l} d_{33}<\kappa >w-d<\mu >\partial_{11}w+d<\rho >\ddot{w}+d_{3}<\kappa \partial g>v=0,\\ d_{3}<\kappa \partial g>w+d[<\kappa {\left(\partial g\right)}^{2}>v-<\mu {\left(g\right)}^{2}>\partial_{11}v+<\rho {\left(g\right)}^{2}>\ddot{v}]=0. \end{array}$$

Solutions to the above equations can be assumed as:

(12) $$w(x,t)=A_{w}\exp[-ik(x+Vt)],\;\;\;v(x,t)=A_{v}\exp[-ik(x+Vt)],$$

where *A_w_*, *A_v_* are amplitudes not equal to zero; *k* is a wave number; and *V* is propagation speed.

#### 4.1.3. Characteristic Equation and Wave Propagation Speeds

Because the obtained results are independent of the amplitude of function *g*, this function can be assumed to satisfy:

(13) $$<\rho {\left(g\right)}^{2}>=h^{2}<\rho >,\;<\mu {\left(g\right)}^{2}>=h^{2}<\mu >,\;<\kappa {\left(g\right)}^{2}>=h^{2}<\kappa >.$$

Substituting (12) into Equation (11), using relations (13) and introducing denotations:

(14) $$\begin{array}{lll} \bar{\kappa }\equiv <\kappa >,\; & {\bar{\kappa }}_{1}\equiv <\kappa \partial g>, & {\bar{\kappa }}_{11}\equiv <\kappa {\left(\partial g\right)}^{2}>,\\ \bar{\mu }\equiv <\mu >, & {\bar{\mu }}_{11}\equiv <\mu {\left(\partial g\right)}^{2}>,\; & \bar{\rho }\equiv <\rho > \end{array}$$

the system of algebraic equations can be obtained:

(15) $$\left[\begin{array}{cc} d_{33}\bar{\kappa }+d(\bar{\mu }-\bar{\rho }V^{2})k^{2} & d_{3}{\bar{\kappa }}_{1}\\ d_{3}{\bar{\kappa }}_{1} & d[{\bar{\kappa }}_{11}+(\bar{\mu }-\bar{\rho }V^{2})h^{2}k^{2}] \end{array}\right]\left\{\begin{array}{c} A_{w}\\ A_{v} \end{array}\right\}=\left\{0\right\}.$$

Introducing denotations:

(16) $$\begin{array}{lll} {\bar{c}}_{1}\equiv d^{2}h^{2}{\bar{\rho }}^{2},\; & {\bar{e}}_{1}\equiv 2d^{2}h^{2}\bar{\rho }\bar{\mu }, & {\bar{e}}_{2}\equiv d\bar{\rho }(d{\bar{\kappa }}_{11}+d_{33}h^{2}\bar{\kappa }),\\ {\bar{f}}_{1}\equiv d^{2}h^{2}{\bar{\mu }}^{2}, & {\bar{f}}_{2}\equiv d\bar{\mu }(d{\bar{\kappa }}_{11}+d_{33}h^{2}\bar{\kappa }),\; & {\bar{f}}_{3}\equiv dd_{33}\bar{\kappa }{\bar{\kappa }}_{11}-{\left(d_{3}\right)}^{2}{{\bar{\kappa }}_{1}}^{2}; \end{array}$$

and also:

(17) $$\bar{c}\equiv {\bar{c}}_{1}k^{4},\;\bar{e}\equiv {\bar{e}}_{1}k^{4}+{\bar{e}}_{2}k^{2},\;\bar{f}\equiv {\bar{f}}_{1}k^{4}+{\bar{f}}_{2}k^{2}+{\bar{f}}_{3},$$

and set the determinant of the system of Equation (15) equal to zero; the characteristic equation takes the form:

(18) $$\bar{c}V^{4}-\bar{e}V^{2}+\bar{f}=0.$$

Solutions to Equation (18) have the form:

(19) $${{\bar{v}}_{-}}^{2}=\frac{1}{2\bar{c}}(\bar{e}-\sqrt{{\bar{e}}^{2}-4\bar{c}\bar{f}}),\;{{\bar{v}}_{+}}^{2}=\frac{1}{2\bar{c}}(\bar{e}+\sqrt{{\bar{e}}^{2}-4\bar{c}\bar{f}}),$$

where ${\bar{v}}_{-}$ is *a lower transverse wave propagation speed*; ${\bar{v}}_{+}$ is *a higher transverse wave propagation speed* in the framework of the tolerance model of periodically layered subsoil on a foundation.

### 4.2. Asymptotic Model

#### 4.2.1. Model Equations for Transverse One-Directional Wave Propagation

Equation (10) takes the form:

(20) $$\begin{array}{l} d_{33}<\kappa >w-d<\mu >\partial_{11}w+d<\rho >\ddot{w}+d_{3}<\kappa \partial g>v=0,\\ d_{3}<\kappa \partial g>w+d<\kappa {\left(\partial g\right)}^{2}>v=0. \end{array}$$

Solutions to Equation (20) can also be assumed in the form (12).

#### 4.2.2. Characteristic Equation and Wave Propagation Speed

Substituting (12) into Equation (20), using relations (13) and (14), the system of algebraic equations of the asymptotic model has the form:

(21) $$\left[\begin{array}{cc} d_{33}\bar{\kappa }+d(\bar{\mu }-\bar{\rho }V^{2})k^{2} & d_{3}{\bar{\kappa }}_{1}\\ d_{3}{\bar{\kappa }}_{1} & d{\bar{\kappa }}_{11} \end{array}\right]\left\{\begin{array}{c} A_{w}\\ A_{v} \end{array}\right\}=\left\{0\right\}.$$

Introducing denotations:

(22) $${\tilde{e}}_{2}\equiv d\bar{\rho }d{\bar{\kappa }}_{11},\;{\tilde{f}}_{2}\equiv d\bar{\mu }d{\bar{\kappa }}_{11},\;{\bar{f}}_{3}\equiv dd_{33}\bar{\kappa }{\bar{\kappa }}_{11}-{\left(d_{3}\right)}^{2}{{\bar{\kappa }}_{1}}^{2};$$

and also:

(23) $$\tilde{e}\equiv {\bar{e}}_{2}k^{2},\;\tilde{f}\equiv {\tilde{f}}_{2}k^{2}+{\bar{f}}_{3},$$

and set the determinant of the system of Equation (21) equal to zero; the characteristic equation takes the form:

(24) $$-\tilde{e}V^{2}+\tilde{f}=0.$$

Solution to Equation (24) has the form:

(25) $${{\tilde{v}}_{-0}}^{2}=\tilde{f}{\tilde{e}}^{-1},$$

where ${\tilde{v}}_{-0}$ is *a transverse wave propagation speed* in the framework of the asymptotic model of periodically layered subsoil on a foundation.

It has to be emphasised that using *the tolerance model of periodically stratified subsoil on a non-deformable foundation, an* additional higher transverse wave propagation speed ${\bar{v}}_{+}$ (19)_2_ is derived, being related to the periodically layered microstructure of the medium. However, in the framework of *the asymptotic model of periodically stratified subsoil on a non-deformable foundation,* only one wave transverse propagation speed ${\tilde{v}}_{-0}$ (25) can be considered. Moreover, it can be shown that this speed ${\tilde{v}}_{-0}$ is a certain approximation of the lower speed ${\bar{v}}_{-}$ (19)_1_ by the tolerance model.

## 5. Results—Transverse Wave Propagation Speeds

### 5.1. Preliminaries

Calculation will be made for dimensionless parameters of wave speeds, which are determined as:

(26) $$\upsilon_{-}\equiv {\bar{v}}_{-}\sqrt{\rho^{\prime }{\kappa^{\prime }}^{-1}},\;\upsilon_{+}\equiv {\bar{v}}_{+}\sqrt{\rho^{\prime }{\kappa^{\prime }}^{-1}},\;\upsilon_{0}\equiv {\tilde{v}}_{-0}\sqrt{\rho^{\prime }{\kappa^{\prime }}^{-1}},$$

where υ___ is *a dimensionless parameter of the lower transverse wave propagation speed*; υ_+_ is *a dimensionless parameter of the higher transverse wave propagation speed* within the tolerance model of a periodically layered medium on a foundation; υ_0_ is *a dimensionless parameter of transverse wave propagation speed* for the asymptotic model of the considered subsoil.

**Figure 3:**
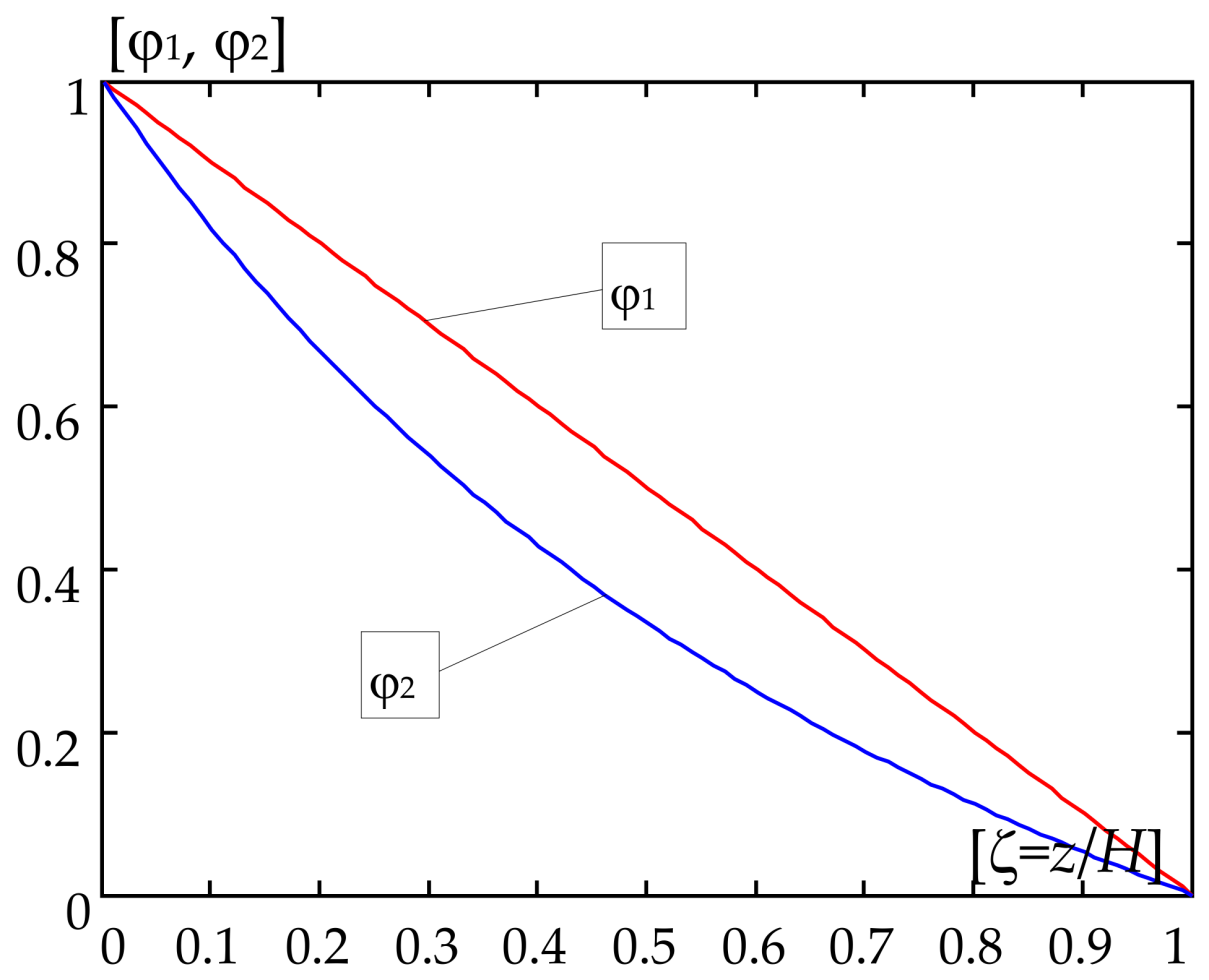
Diagrams of decay functions (27), (28).

Calculations are made for two decay functions:

(27) $$\varphi_{1}(z/H)=1-z/H;$$

(28) $$\varphi_{2}(z/H)=\frac{1-z/H}{1+z/H};$$

where *H* is the thickness of the subsoil. Plots of these functions are shown in Figure 3.

Then, using the formulae (8), the coefficients for these functions have values:

(29) $$\begin{array}{l} d^{1}\equiv \int_{0}^{1}{\varphi_{1}}^{2}d\zeta =0.333,\;d_{3}^{1}\equiv \int_{0}^{1}\varphi_{1}\partial \varphi d\zeta =-0.5,\;d_{33}^{1}\equiv \int_{0}^{1}{\left(\partial \varphi_{1}\right)}^{2}d\zeta =1;\zeta =z/H;\\ d^{2}\equiv \int_{0}^{1}{\varphi_{2}}^{2}d\zeta =0.227,\;d_{3}^{2}\equiv \int_{0}^{1}\varphi_{2}\partial \varphi d\zeta =-0.386,\;d_{33}^{2}\equiv \int_{0}^{1}{\left(\partial \varphi_{2}\right)}^{2}d\zeta =1.167. \end{array}$$

Let us also introduce the following dimensionless parameters:

(30) $$\delta =h/H,\;\varepsilon =E^{\prime \prime }/E^{\prime },\;\varsigma =\rho^{\prime \prime }/\rho^{\prime },$$

where *H* is the thickness of subsoil; *h* is the microstructure size parameter; ρ′, ρ″ are mass densities; and *E*′, *E*″ are Young’s moduli. The parameter δ is a dimensionless microstructure parameter; ε is a parameter determining differences in Young’s moduli; and ς is a parameter determining differences in densities.

### 5.2. Diagrams of Results

**Figure 4:**
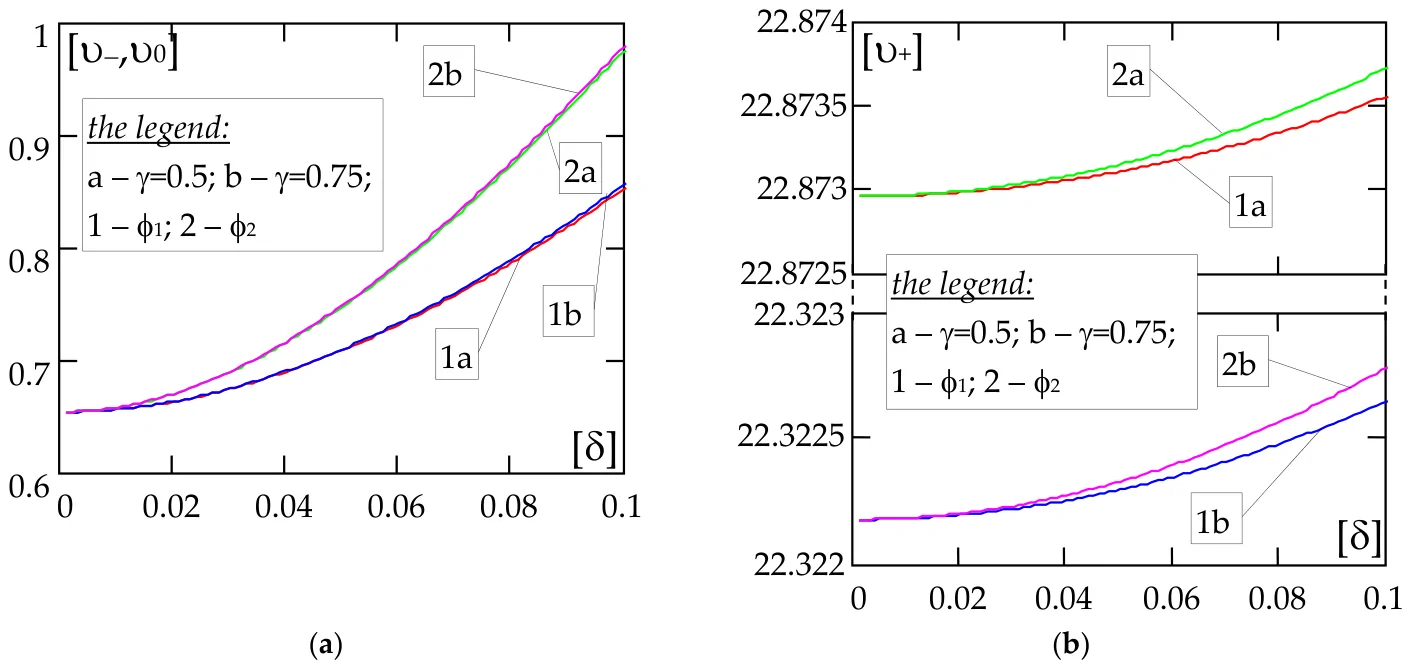

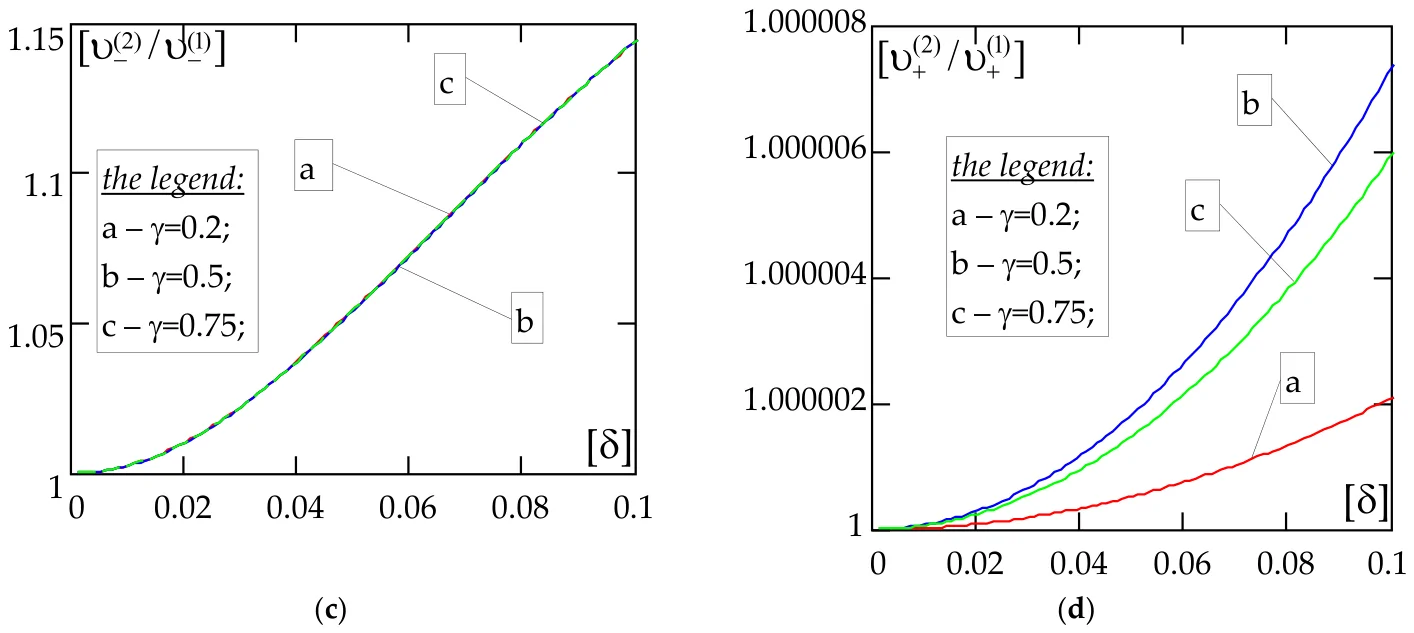
Diagrams for dimensionless parameters of transverse wave propagation speeds as functions of the dimensionless microstructure parameter δ (for fixed ratios: ε = ς = 0.5; and for γ = (0.2; 0.5; 0.75)): (**a**) lower speeds υ_0_, υ_−_; (**b**) higher speeds υ_+_ (shown for two intervals of vertical axis υ_+_); (**c**) ratios of lower speeds for two decay functions (27), (28) $\upsilon_{-}^{\left(2\right)}/\upsilon_{-}^{\left(1\right)}$; (**d**) ratios of higher speeds for two decay functions (27), (28) $\upsilon_{+}^{\left(2\right)}/\upsilon_{+}^{\left(1\right)}$; (indexes “1”, “2” denote parameters for decay functions (27), (28), respectively).

**Figure 5:**
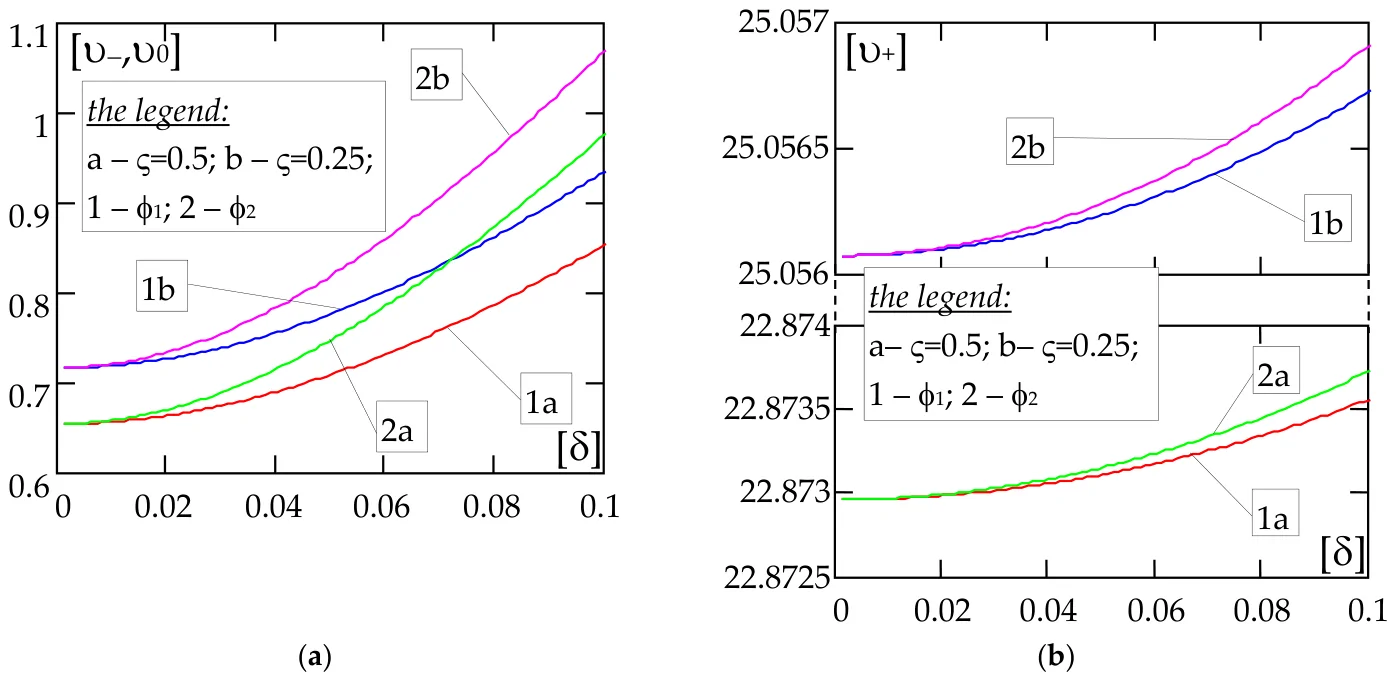

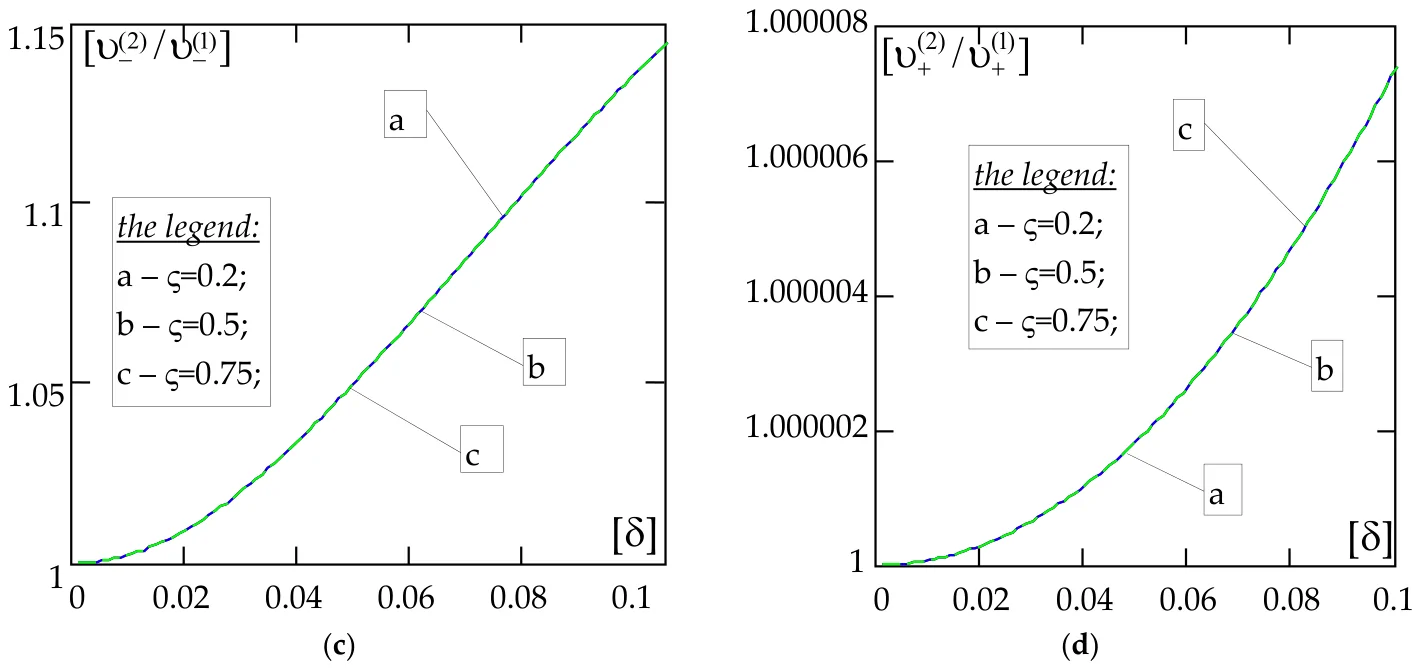
Diagrams for dimensionless parameters of transverse wave propagation speeds as functions of the dimensionless microstructure parameter δ (for fixed ratios: ε = γ = 0.5; and for ς = (0.2; 0.25; 0.5; 0.75)): (**a**) lower speeds υ_0_, υ_−_; (**b**) higher speeds υ_+_ (shown for two intervals of vertical axis υ_+_); (**c**) ratios of lower speeds for two decay functions (27), (28) $\upsilon_{-}^{\left(2\right)}/\upsilon_{-}^{\left(1\right)}$; (**d**) ratios of higher speeds for two decay functions (27), (28) $\upsilon_{+}^{\left(2\right)}/\upsilon_{+}^{\left(1\right)}$; (indexes “1”, “2” denote parameters for decay functions (27), (28), respectively).

**Figure 6:**
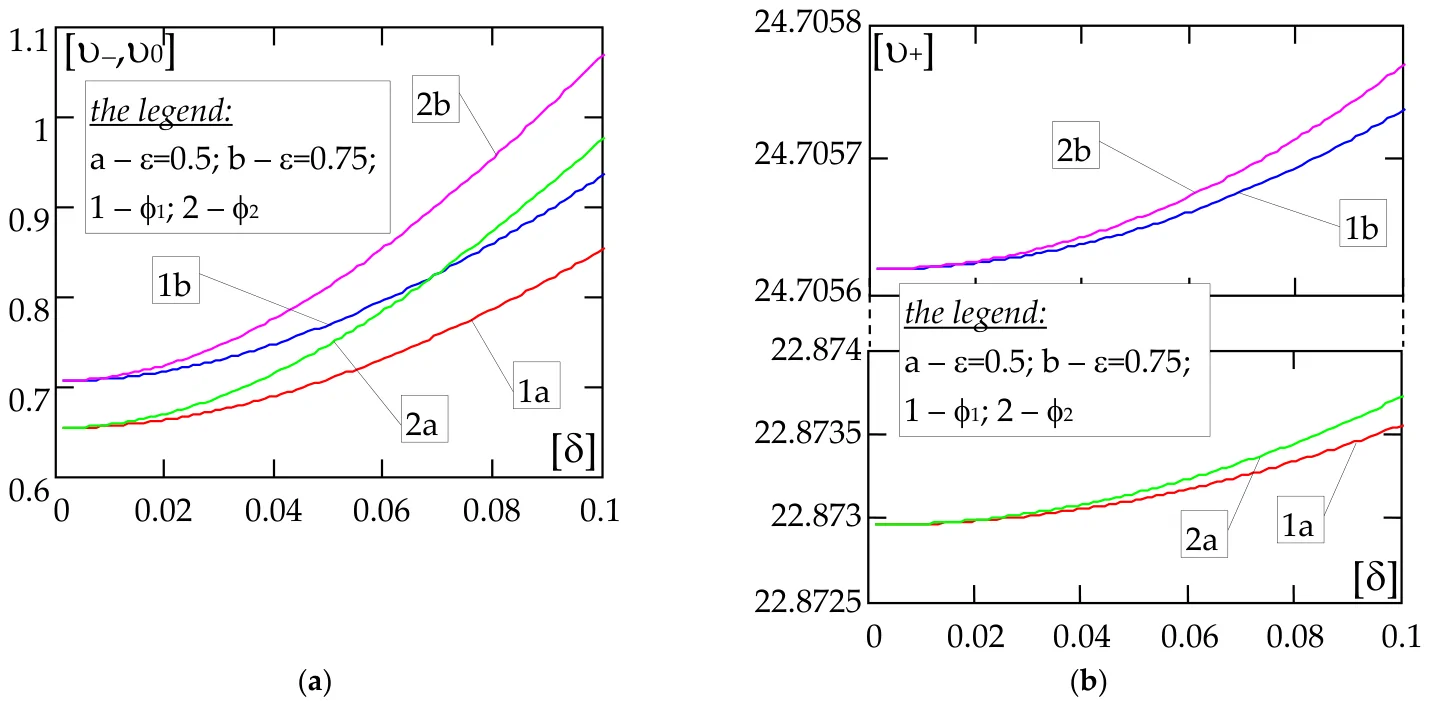

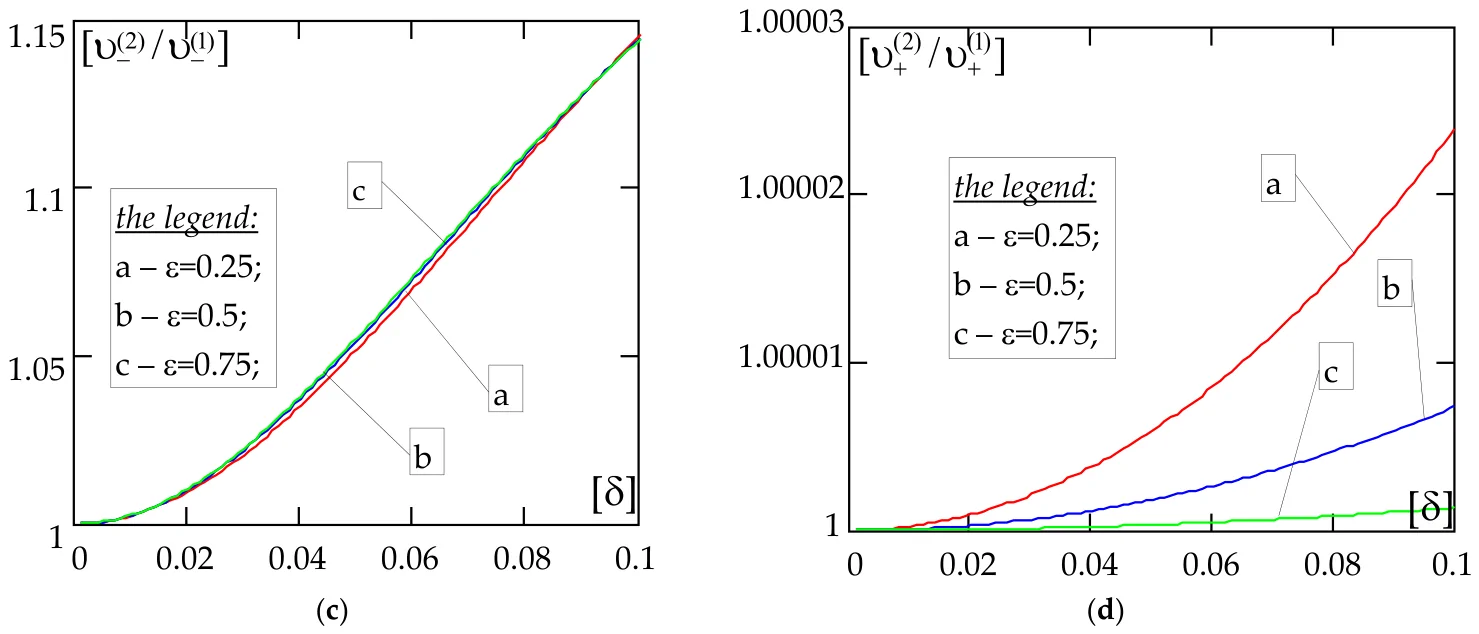
Diagrams for dimensionless parameters of transverse wave propagation speeds as functions of the dimensionless microstructure parameter δ (for fixed ratios: ς = γ = 0.5; and for ε = (0.25; 0.5; 0.75)): (**a**) lower speeds υ_0_, υ_−_; (**b**) higher speeds υ_+_ (shown for two intervals of vertical axis υ_+_); (**c**) ratios of lower speeds for two decay functions (27), (28) $\upsilon_{-}^{\left(2\right)}/\upsilon_{-}^{\left(1\right)}$; (**d**) ratios of higher speeds for two decay functions (27), (28) $\upsilon_{+}^{\left(2\right)}/\upsilon_{+}^{\left(1\right)}$; (indexes “1”, “2” denote parameters for decay functions (27), (28), respectively).

**Figure 7:**
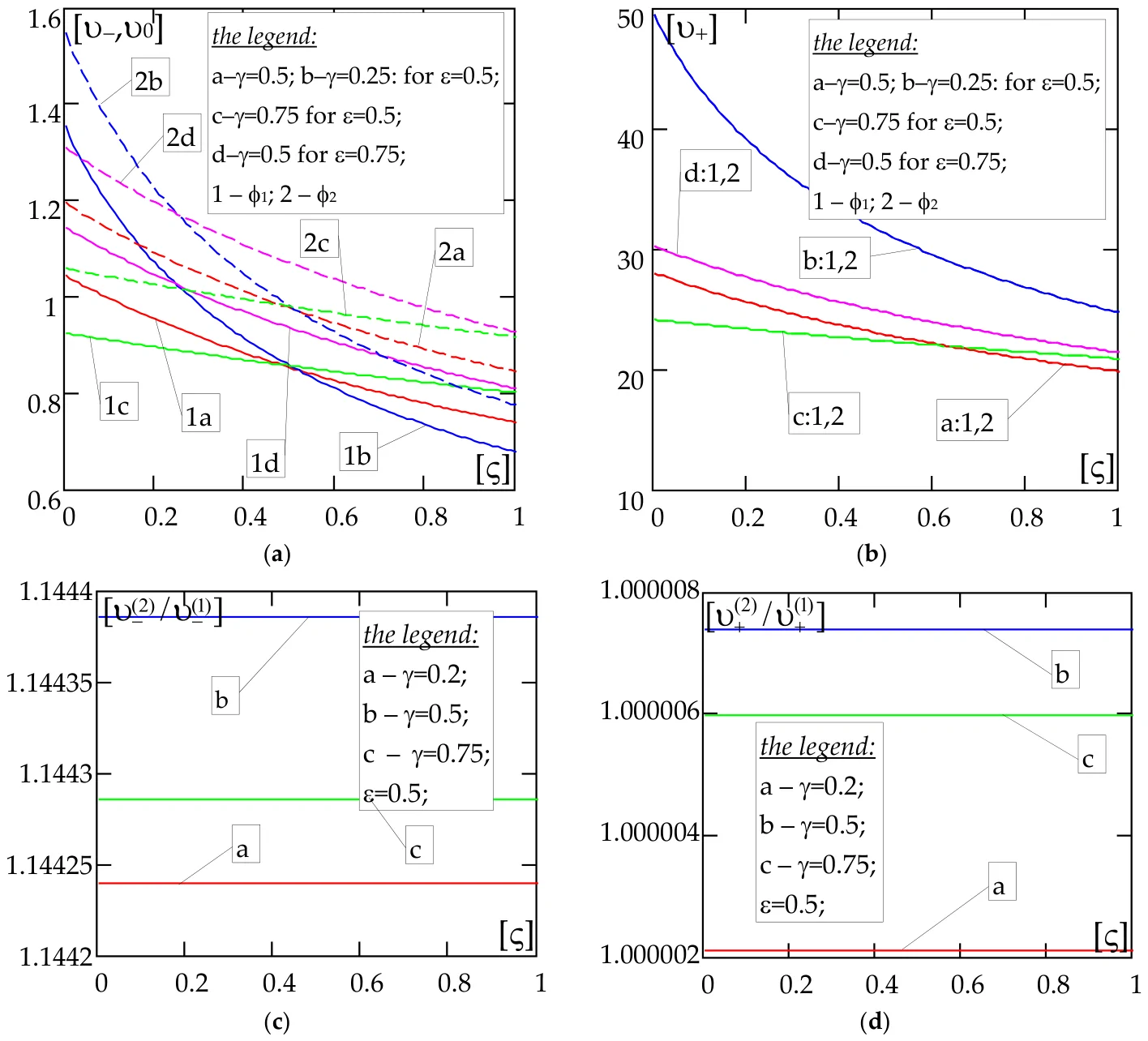
Diagrams for dimensionless parameters of transverse wave propagation speeds as functions of the dimensionless parameter ς, determining differences in mass densities (for fixed ratio: δ = 0.1; and for γ = (0.2; 0.25; 0.5; 0.75), ε = (0.5; 0.75)): (**a**) lower speeds υ_0_, υ_−_; (**b**) higher speeds υ_+_; (**c**) ratios of lower speeds for two decay functions (27), (28) $\upsilon_{-}^{\left(2\right)}/\upsilon_{-}^{\left(1\right)}$; (**d**) ratios of higher speeds for two decay functions (27), (28) $\upsilon_{+}^{\left(2\right)}/\upsilon_{+}^{\left(1\right)}$; (indexes “1”, “2” denote parameters for decay functions (27), (28), respectively).

**Figure 8:**
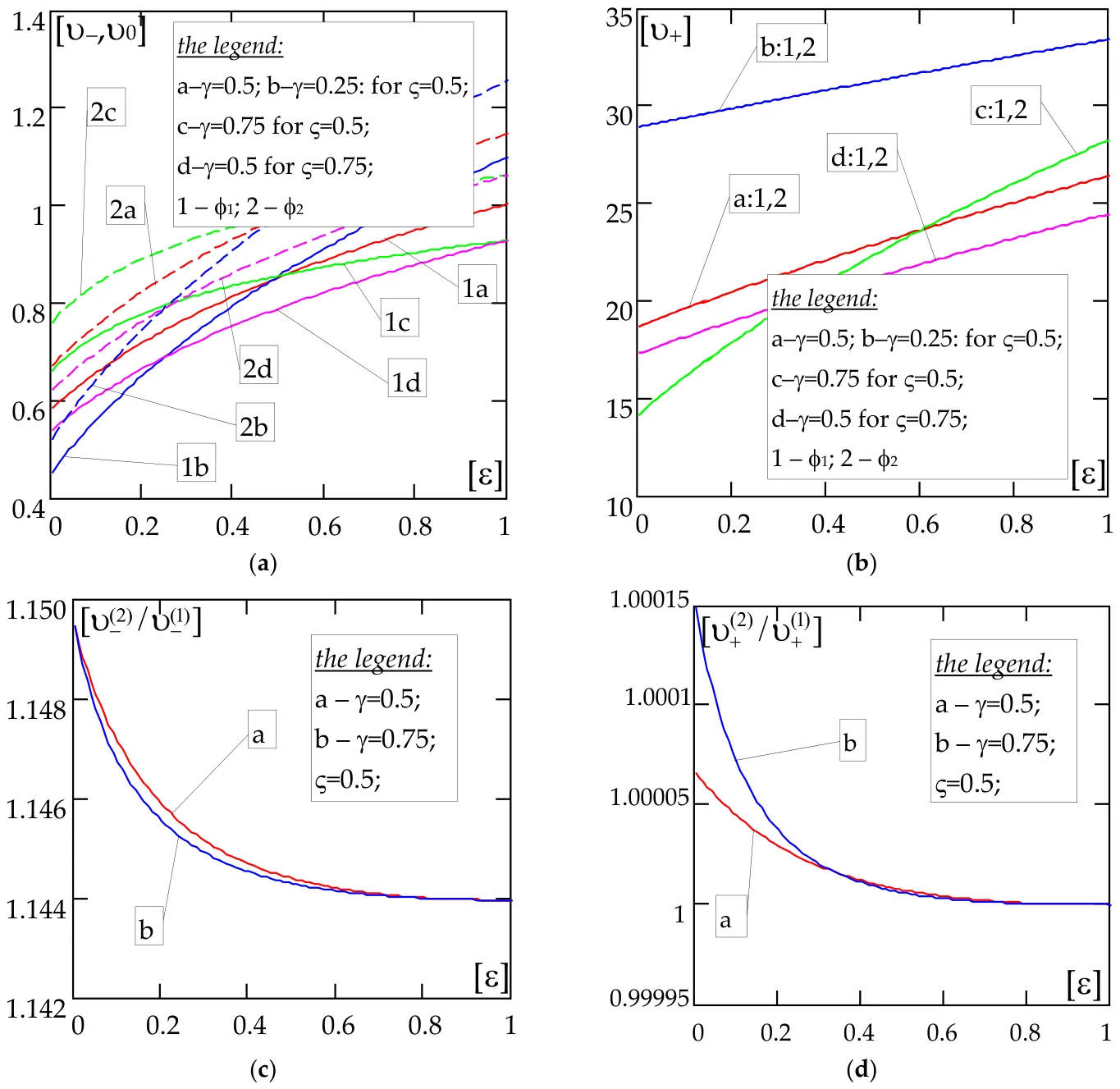
Diagrams for dimensionless parameters of transverse wave propagation speeds as functions of the dimensionless parameter ε, determining differences in Young’s moduli (for fixed ratio: δ = 0.1; and for γ = (0.25; 0.5; 0.75), ς = (0.5; 0.75)): (**a**) lower speeds υ_0_, υ_−_; (**b**) higher speeds υ_+_; (**c**) ratios of lower speeds for two decay functions (27), (28) $\upsilon_{-}^{\left(2\right)}/\upsilon_{-}^{\left(1\right)}$; (**d**) ratios of higher speeds for two decay functions (27), (28) $\upsilon_{+}^{\left(2\right)}/\upsilon_{+}^{\left(1\right)}$; (indexes “1”, “2” denote parameters for decay functions (27), (28), respectively).

**Figure 9:**
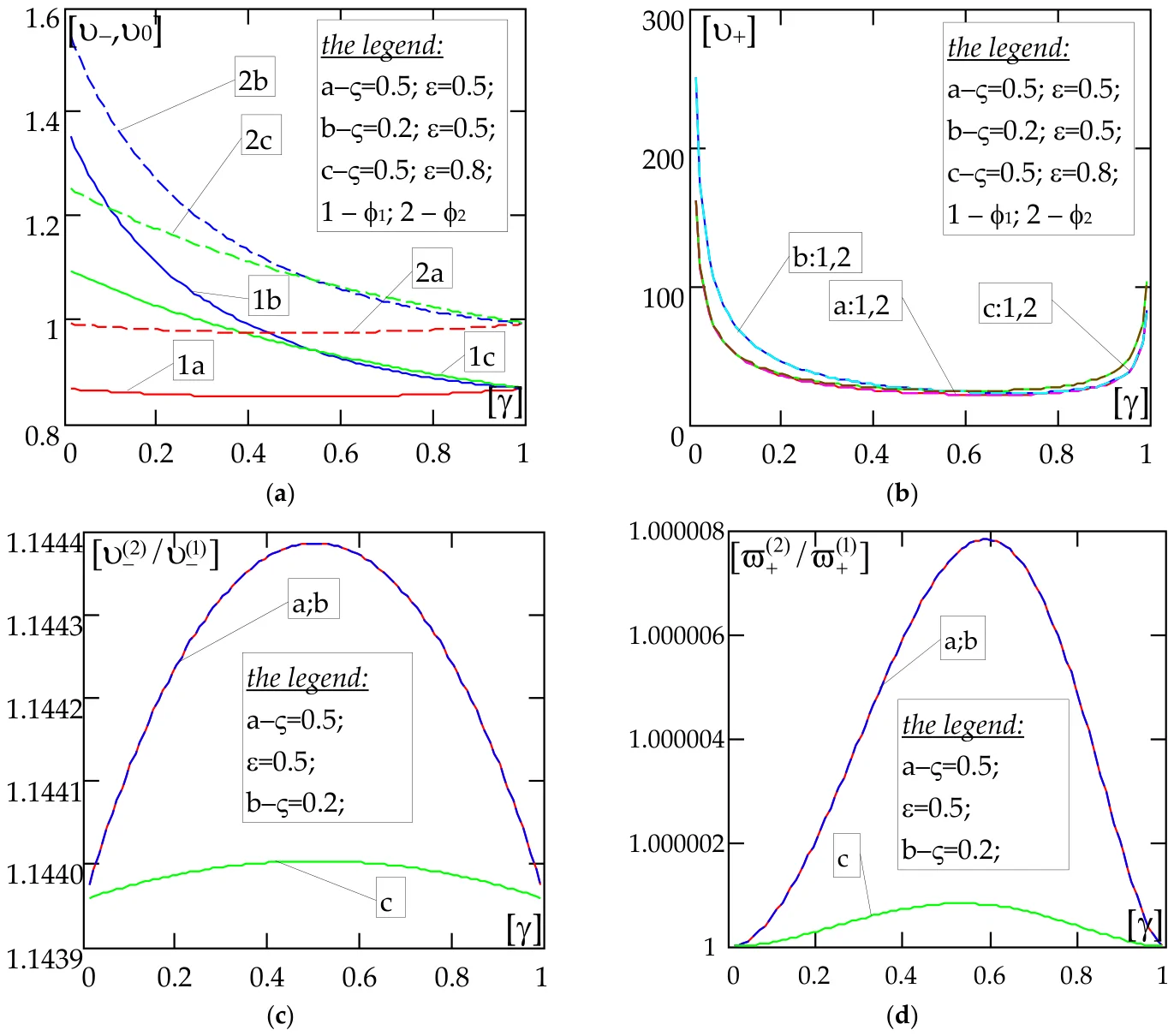
Diagrams for dimensionless parameters of transverse wave propagation speeds as functions of the dimensionless parameter γ, being the non-homogeneity ratio (for fixed ratio: δ = 0.1; and for ς = (0.2; 0.5), ε = (0.5; 0.8)): (**a**) lower speeds υ_0_, υ_−_; (**b**) higher speeds υ_+_; (**c**) ratios of lower speeds for two decay functions (27), (28) $\upsilon_{-}^{\left(2\right)}/\upsilon_{-}^{\left(1\right)}$; (**d**) ratios of higher speeds for two decay functions (27), (28) $\upsilon_{+}^{\left(2\right)}/\upsilon_{+}^{\left(1\right)}$; (indexes “1”, “2” denote parameters for decay functions (27), (28), respectively).

Results of calculations will be presented in the form of plots in Figure 4, Figure 5, Figure 6, Figure 7, Figure 8 and Figure 9.

Plots in Figure 4, Figure 5, Figure 6, Figure 7, Figure 8 and Figure 9 are made for two cases “1” and “2”, related to two different decay functions, (27) and (28).

The graphs show the results for dimensionless velocity parameters or their ratios as a function of: the dimensionless microstructure parameter δ (Figure 4, Figure 5 and Figure 6); the dimensionless parameter ς, which defines the difference in density, Figure 7; the dimensionless parameter ε, which defines the difference in Young’s moduli, Figure 8; and the dimensionless parameter γ, which is the non-homogeneity ratio, Figure 9.

In all calculations, it is assumed that the Poisson’s ratio is fixed as ν = 0.3.

Figure 4, Figure 5 and Figure 6 present dimensionless velocity parameters or their ratios as a function of the dimensionless microstructure parameter δ. Results shown in Figure 4 are calculated for fixed values of parameters: ε = ς = 0.5. Figure 4a,b present the dimensionless parameters for lower speeds υ_0_, υ_−_ and higher speeds υ_+_, respectively, made for the parameter γ = 0.5; 0.75. Moreover, Figure 4c,d show the ratios of dimensionless parameters of two different decay functions φ_1_, (27), and φ_2_, (28), for lower speeds $\upsilon_{-}^{\left(2\right)}/\upsilon_{-}^{\left(1\right)}$ and higher speeds $\upsilon_{+}^{\left(2\right)}/\upsilon_{+}^{\left(1\right)}$, respectively, made for the parameter γ = 0.2; 0.5; 0.75.

From plots shown in Figure 4a,b, it can be observed that the parameters of lower, υ_0_, υ_−_, and higher speeds, υ_+_, respectively, increase with increasing the dimensionless microstructure parameter δ. The decay functions have an effect on lower and higher speeds—the parameters of lower and higher speeds for the first decay function φ_1_, (27), are smaller than for the function φ_2_, (28), cf. Figure 4a,b. These differences between the parameters of speeds are greater for lower speeds than for higher speeds. For greater parameter γ values, the parameters of lower speeds are higher (Figure 4a), but of higher speeds are smaller (Figure 4b). Differences between lower speeds for the two considered decay functions (Figure 4c) are nearly identical for various parameters, γ; however, between higher speeds (Figure 4d) they are noticeable but very small. Moreover, these differences increase with increasing the microstructure parameter δ. It is also worth noting that the dimensionless parameters for lower speeds—in the tolerance model υ_−_ and the asymptotic model υ_0_ are nearly identical, Figure 4a.

Results shown in Figure 5 are calculated for fixed values of parameters: ε = γ = 0.5. Figure 5a,b present the dimensionless parameters for lower speeds υ_0_, υ_−_ and higher speeds υ_+_, respectively, made for the parameter ς = 0.25; 0.5. Moreover, Figure 5c,d show the ratios of dimensionless parameters of the two decay functions φ_1_, (27), and φ_2_, (28), for lower speeds $\upsilon_{-}^{\left(2\right)}/\upsilon_{-}^{\left(1\right)}$ and higher speeds $\upsilon_{+}^{\left(2\right)}/\upsilon_{+}^{\left(1\right)}$, respectively, made for the parameter ς = 0.2; 0.5; 0.75.

From the plots shown in Figure 5a,b, an identical comment can be formulated about the dependency of the parameters of lower, υ_0_, υ_−_, and higher speeds, υ_+_, respectively, from the dimensionless microstructure parameter δ. The decay functions have an effect on lower and higher speeds—the parameters of lower and higher speeds for the first decay function φ_1_, (27), are smaller than for the function φ_2_, (28), cf. Figure 5a,b.

These differences between the parameters of speeds are greater for lower speeds than for higher speeds. For greater parameter ς values, the parameters of lower speeds and higher speeds are smaller (Figure 5a,b). Differences between lower and higher speeds for the two considered decay functions (Figure 5c,d) are nearly identical for various parameters ς. Moreover, these differences increase with increasing the microstructure parameter δ.

Results shown in Figure 6 are calculated for fixed values of parameters: γ = ς = 0.5. Figure 6a,b present the dimensionless parameters for lower speeds υ_0_, υ_−_ and higher speeds υ_+_, respectively, made for the parameter ε = 0.5; 0.75. Moreover, Figure 6c,d show the ratios of dimensionless parameters of two various decay functions φ_1_, (27), and φ_2_, (28), for lower speeds $\upsilon_{-}^{\left(2\right)}/\upsilon_{-}^{\left(1\right)}$ and higher speeds $\upsilon_{+}^{\left(2\right)}/\upsilon_{+}^{\left(1\right)}$, respectively, made for the parameter γ = 0.25; 0.5; 0.75.

From plots shown in Figure 6a,b, it can be observed that the decay functions have an effect on lower and higher speeds—the parameters of lower and higher speeds for the first decay function φ_1_, (27), are smaller than for the function φ_2_, (28), cf. Figure 6a,b. These differences between the parameters of speeds are greater for lower speeds than for higher speeds. For greater parameter ε values, the parameters of lower speeds and higher speeds are higher, Figure 6a,b, respectively. Differences between lower speeds (Figure 6c) or higher speeds (Figure 6d) for the two considered decay functions are noticeable but very small. For smaller parameter ε, the differences in lower speeds (Figure 6c) are smaller, but of higher speeds (Figure 6d) are greater.

Results shown in Figure 7 are calculated for a fixed value of the parameter: δ = 0.1. Figure 7a,b present the dimensionless parameters for lower speeds υ_0_, υ_−_ and higher speeds υ_+_, respectively, as functions of the parameter ς, made for the parameters ε = 0.5; 0.75 and γ = 0.25; 0.5; 0.75. Moreover, Figure 7c,d, show the ratios of dimensionless parameters of the two decay functions φ_1_, (27), and φ_2_, (28), for lower speeds $\upsilon_{-}^{\left(2\right)}/\upsilon_{-}^{\left(1\right)}$ and higher speeds $\upsilon_{+}^{\left(2\right)}/\upsilon_{+}^{\left(1\right)}$, respectively, versus the parameter γ, made for the parameters: ε = 0.5 and γ = 0.2; 0.5; 0.75.

From the plots shown in Figure 7a,b, it can be observed that the decay functions have an effect on lower and higher speeds—the parameters of these speeds for the first decay function φ_1_, (27), are smaller than for the function φ_2_, (28), cf. Figure 7a,b, but higher speeds are nearly identical for the same decay function. These differences between the parameters of speeds are greater for lower speeds than for higher speeds, Figure 7c,d. They are also nearly independent of ς.

Results presented in Figure 8 are calculated for a fixed value of the parameter: δ = 0.1. Figure 8a,b show the dimensionless parameters for lower speeds υ_0_, υ_−_ and higher speeds υ_+_, respectively, as functions of the parameter ε, made for the parameters: ς = 0.5; 0.75 and γ = 0.25; 0.5; 0.75. Moreover, Figure 8c,d present the ratios of dimensionless parameters of the two decay functions φ_1_, (27), and φ_2_, (28), for lower speeds $\upsilon_{-}^{\left(2\right)}/\upsilon_{-}^{\left(1\right)}$ and higher speeds $\upsilon_{+}^{\left(2\right)}/\upsilon_{+}^{\left(1\right)}$, respectively, versus the parameter γ, made for the parameters: ς = 0.5 and γ = 0.5; 0.75.

It can be observed that the parameters of lower and higher speeds for the first decay function φ_1_, (27), are smaller than for the function φ_2_, (28), cf. Figure 8a,b. Moreover, higher speeds are nearly identical for the same decay function. These differences between the parameters of speeds are greater for lower speeds than for higher speeds, Figure 8c,d. For greater parameter γ, the differences in lower speeds are smaller, but the differences in higher speeds are greater. Moreover, these differences tend to 1 for ε tending to 1, Figure 8c,d.

Results shown in Figure 9 are calculated for a fixed value of the parameter: δ = 0.1. Figure 9a,b present the dimensionless parameters for lower speeds υ_0_, υ_−_ and higher speeds υ_+_, respectively, as functions of the parameter γ, made for the parameters: ς = 0.2; 0.5 and ε = 0.5; 0.8. Moreover, Figure 9c,d show the ratios of dimensionless parameters of the two decay functions φ_1_, (27), and φ_2_, (28), for lower speeds $\upsilon_{-}^{\left(2\right)}/\upsilon_{-}^{\left(1\right)}$ and higher speeds $\upsilon_{+}^{\left(2\right)}/\upsilon_{+}^{\left(1\right)}$, respectively, versus the parameter γ, made for the parameters: ς = 0.2; 0.5 and ε = 0.5; 0.8.

It can be observed that the parameters of lower and higher speeds for the first decay function φ_1_, (27), are smaller than for the function φ_2_, (28), cf. Figure 9a,b. Moreover, higher speeds are nearly identical for the same decay function, Figure 9b. These differences between the parameters of speeds are very small. It can be noticed that changing parameter ς has practically no effect on the differences, but for greater parameter ε, the differences in speeds are smaller, cf. Figure 9c,d.

## 6. Final Comments

This paper analyses the propagation of waves travelling through a periodically laminated medium resting on an undeformable substrate.

Due to the periodicity of the object, the equations describing this problem are differential equations with highly oscillating, discontinuous functional coefficients in the direction perpendicular to the layering. Equations in this form are not a suitable tool for analysing the selected problems. In order to obtain a better tool for analysing these cases, the tolerance modelling procedure [1,56,57] was applied in this article to the equations mentioned above. The tolerance method transforms equations with highly oscillating functional coefficients into differential equations with constant coefficients. The equations thus obtained constitute the tolerance model equations for wave propagation in the medium under consideration. The resulting equations describe the influence of microstructure size on this problem, through the dependence of certain coefficients on the so-called microstructure size parameter (cell-layer height).

In this study, the equations of the tolerance model were subsequently applied to analyse the propagation speed of a transverse wave (surface wave) in a single direction. The direction of wave propagation was assumed to be parallel to the laminations (along the *x*_1_ = *x* axis), with the displacement component perpendicular to the laminations (along the *x*_3_ = *z* axis). In this example, the effect of variations in Young’s moduli and mass densities within the periodic cell was analysed, as well as the microstructure size effect and the non-homogeneity ratio on the values of the lower and higher wave propagation velocities.

On the basis of the above considerations, a number of general comments can be made.

1. *The tolerance method* allows for replacing equations with functional, highly oscillating, discontinuous coefficients—describing a periodically laminated object—by equations with constant coefficients, known as *the tolerance model*.

2. The equations of the tolerance model allow for investigating the microstructure parameter effect on, amongst other things, wave propagation in the medium under analysis, as they include terms with coefficients dependent on the microstructure size parameter *h* (laminate thickness).

3. Applying *the tolerance model* to the problem of unidirectional transverse wave propagation in a periodically laminated medium yields two velocities: a fundamental, lower propagation speed associated with the macrostructure of the medium, and a higher propagation speed associated with the microstructure of the medium.

4. This paper demonstrates another application of *the tolerance method* to the analysis of thermomechanical problems in micro-inhomogeneous structures, in this case: to the propagation of waves in periodically laminated media on a rigid substrate. *The tolerance method* is quite a universal tool for investigating structures of this type.

5. Asymptotic model equations were introduced for comparing the results; these allow only the fundamental lower propagation velocities to be considered.

However, based on the presented calculation results for the two decay functions, the following comments can be made.

1. Both velocities of the transverse wave propagation under consideration—the lower and the higher—depend on the microstructure parameter, with the dependence of the lower speeds being stronger (more visible in the graphs).

2. The influence of different decay functions on the speeds can be observed; in the example under consideration, both the lower and higher speeds are lower for decay function No. 1. Greater differences between the speeds occur in the case of the lower speeds, Figure 4, Figure 5, Figure 6, Figure 7, Figure 8 and Figure 9.

3. The effects of the microstructure parameter and the form of the decay function on the velocities also depend on the values of the following parameters: ε–determining the differences in Young’s moduli, ς–determining the differences in mass densities, and γ–the non-homogeneity parameter.

4. Lower and higher speeds depend on the density parameter ς and the stiffness parameter ε in a manner known from the literature, i.e., these speeds decrease with an increase in the density parameter and with a decrease in the stiffness parameter; cf. Figure 7 and Figure 8. However, in the case of lower velocities, there are noticeable differences in their values calculated for different decay functions, whereas for higher velocities the influence of these functions is practically negligible.

5. From the graphs in Figure 9, it can be seen that both speeds depend on the non-homogeneity parameter γ, but in different ways; this also depends on other parameters, such as the density parameter ς and the stiffness parameter ε. According to the results presented, the lower velocities decrease as the heterogeneity parameter γ increases, but, for example, for the set of parameters ς = ε = 0.5, they first decrease slowly and then increase slowly. Higher velocities, on the other hand, first decrease strongly with an increase in the heterogeneity parameter γ, then decrease slowly, and finally increase rapidly at the end of the interval [0;1].

6. As a means of verifying the calculations using the tolerance model, an asymptotic model was employed, which does not allow for the analysis of higher speeds, but only lower ones. The values of the lower speeds according to the tolerance model and the asymptotic model are almost identical (the curves are practically indistinguishable).

In summary, it can be concluded that the tolerance method is universal, as it can be applied to the modelling of various micro-inhomogeneous structures. In subsequent studies on the laminates presented here, more complex cases of wave propagation will be examined, taking into account additional displacement components and analysing additional propagation velocities.

## Figures and Tables

**Table 1:** List of key symbols.

No.	Symbol	Comment
1.	<·>	the averaging operator
2.	α, β, …	subscripts run over 1, 2
3.	*A*, *B*, …	superscripts run over 1,…,*N*
4.	*A_w_*, *A_v_*	the amplitudes of the components *w*, *v*
5.	χ	the small parameter (χ << 1), called the tolerance parameter
6.	$\bar{c},\;\bar{e},\;\bar{f}$	coefficients of the characteristic Equation (18)
7.	$\tilde{e},\;\tilde{f}$	coefficients of the characteristic Equation (24)
8.	${\tilde{e}}_{2},\;{\tilde{f}}_{2}\;{\tilde{f}}_{3}$	components of coefficients (23), $\tilde{e},\;\tilde{f}$
9.	*c_ijkl_* = *c_ijkl_*(*z*)	elastic moduli of the medium
10.	${c^{\prime }}_{ijkl},\;{c^{\prime \prime }}_{ijkl}$	components of elasticity tensors of sub-laminas
11.	δ	a dimensionless microstructure parameter, δ=h/H,
12.	Δ	the periodicity cell of a periodic medium, Δ ≡ (−*h*/2,*h*/2) × {**0**}
13.	∂*_i_*, ∂_α_, ∂,	derivatives respected to *x_i_*, *x*_α_, *z*
14.	*d*, *d*_3_, *d*_33_	denotations of certain coefficients related to the decay function φ(*z*)
15.	ε	a parameter determining differences in Young’s moduli, $\varepsilon =E^{\prime \prime }/E^{\prime },$
16.	*e_ij_*	deformations
17.	φ(*z*)	the known decay function, satisfying the conditions φ(0) = 1, φ(*H*) = 0, φ_,3_ < 0,
18.	γ = ν′	a constant parameter, called the non-homogeneity ratio
19.	*g* = *g*(*z*)	the fluctuation shape functions of the 1st kind, $g\in FS^{1}(\Delta ,\chi )$,
20.	*h*	the thickness of laminas, called the microstructure size parameter; *h* << *H*
21.	*h*′, *h*″	sub-laminas thicknesses
22.	*H*	the medium thickness along the *z* axis
23.	*i*, *j*, …	subscripts run over 1, 2, 3
24.	*k*	a wave number
25.	ν′, ν″	material volume fractions in laminas, defined as ν′ = *h*′/*h*, ν″ = *h*″/*h*
26.	λ, μ	the Lame moduli of the medium
27.	λ′, μ′; λ″, μ″	the Lame moduli of sub-laminas
28.	Ω	the region of a laminated subsoil, Ω ≡ (0,*H*) × Π
29.	Π	a region on the plane 0*x*_1_*x*_2_
30.	*p_i_* = *p_i_*(**x**,*t*)	loadings in the *x_i_*-axis directions on the upper surface Π of the subsoil
31.	ρ = ρ(*z*)	mass density of the medium
32.	ρ′, ρ″	mass densities of sub-laminas
33.	$r_{i}(x,z,t)$	a residual field *r_i_*(·)
34.	ς	a parameter determining differences in densities, $\varsigma =\rho^{\prime \prime }/\rho^{\prime }$
35.	*s_ij_*	stresses
36.	*t*	the time coordinate
37.	ϑ	the highly oscillating function, $\vartheta \in HO^{1}(\Delta ,\chi )$, is a function $\vartheta \in H^{1}(\Lambda )$
38.	θ = θ(*z*)	the tolerance-periodic function $\theta \in H^{k}(\Lambda )$, $\theta \in TP^{k}(\Delta ,\chi )$, for *k* = 0,1
39.	*u_i_*	displacements
40.	*w* = *w*_3_; *v* = *v*_3_	components along the *x*_3_ axis
41.	*w_i_*(**x**,*t*)	new unknowns, independent on *z* components of the macrodisplacements *W_i_*
42.	*W_i_*(**x**,*z*,*t*)	the macrodisplacements, *W_i_*(**x**,·,*t*) ∈ *SV*^1^(Δ,χ)
43.	*v_i_*(**x**,*t*)	new unknowns, independent on *z* components of the fluctuation amplitudes *V_i_*
44.	*V_i_*(**x**,*z*,*t*)	the fluctuation amplitudes, *V_i_*(**x**,·,*t*) ∈ *SV*^1^(Δ,χ)
45.	*V*	a propagation speed
46.	${\tilde{v}}_{-0}$	a transverse wave propagation speed by the asymptotic model
47.	${\bar{v}}_{-}$	a lower transverse wave propagation speed by the tolerance model
48.	${\bar{v}}_{+}$	a higher transverse wave propagation speed by the tolerance model
49.	υ_0_	a dimensionless parameter of transverse wave propagation speed
50.	υ___	a dimensionless parameter of lower transverse wave propagation speed
51.	υ_+_	a dimensionless parameter of higher transverse wave propagation speed
52.	Ξ	the slowly varying function, $\Xi \in SV^{1}(\Delta ,\chi )$, is a function $\Xi \in H^{1}(\Lambda )$
53.	*x*	*x*_1_ coordinate
54.	*x_i_*	axes of the system of coordinates
55.	*z*	*x*_3_ coordinate

## Data Availability

The original contributions presented in this study are included in the article. Further inquiries can be directed to the corresponding author.
